# Traditional plant use in Burkina Faso (West Africa): a national-scale analysis with focus on traditional medicine

**DOI:** 10.1186/1746-4269-11-9

**Published:** 2015-02-19

**Authors:** Alexander Zizka, Adjima Thiombiano, Stefan Dressler, Blandine MI Nacoulma, Amadé Ouédraogo, Issaka Ouédraogo, Oumarou Ouédraogo, Georg Zizka, Karen Hahn, Marco Schmidt

**Affiliations:** Institute for Biological and Environmental Sciences, University of Gothenburg, Carl Skottsbergs gata 22B, Box 461, SE 405 30 Göteborg, Sweden; Department of Botany and Molecular Evolution, Senckenberg Research Institute, Senckenberganlage 25, 60325 Frankfurt am Main, Germany; Département de Biologie et Physiologie végétales, Laboratoire de Biologie et Ecologie Végétales, Université de Ouagadougou, 03 BP 7021, Ouagadougou 03, Burkina Faso; Institute for Ecology, Evolution and Diversity, Goethe University Frankfurt, Max-von-Laue-Str. 13, 60438 Frankfurt am Main, Germany; Biodiversity and Climate Research Centre, Senckenberganlage 25, 60325 Frankfurt am Main, Germany

**Keywords:** Ethnobotany, Medicinal plants, Traditional medicine, Economic botany, Usefulness, Relative importance index, West Africa

## Abstract

**Background:**

The West African country of Burkina Faso (BFA) is an example for the enduring importance of traditional plant use today. A large proportion of its 17 million inhabitants lives in rural communities and strongly depends on local plant products for their livelihood. However, literature on traditional plant use is still scarce and a comprehensive analysis for the country is still missing.

**Methods:**

In this study we combine the information of a recently published plant checklist with information from ethnobotanical literature for a comprehensive, national scale analysis of plant use in Burkina Faso. We quantify the application of plant species in 10 different use categories, evaluate plant use on a plant family level and use the relative importance index to rank all species in the country according to their usefulness. We focus on traditional medicine and quantify the use of plants as remedy against 22 classes of health disorders, evaluate plant use in traditional medicine on the level of plant families and rank all species used in traditional medicine according to their respective usefulness.

**Results:**

A total of 1033 species (50%) in Burkina Faso had a documented use. Traditional medicine, human nutrition and animal fodder were the most important use categories. The 12 most common plant families in BFA differed considerably in their usefulness and application. Fabaceae, Poaceae and Malvaceae were the plant families with the most used species. In this study *Khaya senegalensis, Adansonia digitata* and *Diospyros mespiliformis* were ranked the top useful plants in BFA. Infections/Infestations, digestive system disorders and genitourinary disorders are the health problems most commonly addressed with medicinal plants. Fabaceae, Poaceae, Asteraceae, Apocynaceae, Malvaceae and Rubiaceae were the most important plant families in traditional medicine. *Tamarindus indica, Vitellaria paradoxa* and *Adansonia digitata* were ranked the most important medicinal plants.

**Conclusions:**

The national-scale analysis revealed systematic patterns of traditional plant use throughout BFA. These results are of interest for applied research, as a detailed knowledge of traditional plant use can a) help to communicate conservation needs and b) facilitate future research on drug screening.

**Electronic supplementary material:**

The online version of this article (doi:10.1186/1746-4269-11-9) contains supplementary material, which is available to authorized users.

## Background

Burkina Faso (BFA) is a landlocked country in central West Africa, covering an area of 274,000 km^2^. Large parts of the population of BFA live in rural communities [1] and strongly depend on traditional plant products for their daily life [2–5]. Some of the plant species traditionally used in BFA are of regional and global economic importance (e.g. *Adansonia digitata, Parkia biglobosa, Sclerocarya birrea, Tamarindus indica, Vitellaria paradoxa*).

While the connection between useful plants and the daily-life products derived from them is mostly dissolving in modern societies, this link remains much clearer in many rural communities, where traditional plant use often is essential for multiple parts of the daily life. This includes the use of crop plants as food for humans and livestock, the use of woody plant parts for fuel, construction or tool manufacture as well as the application of plants in traditional medicine and for religious purposes. In many cases the traditional use of plants is closely linked to considerable floristic knowledge and appreciation of the used species, not seldom in a spiritual-mystical way [2].

The traditional plant use around the globe represents an invaluable reservoir of knowledge and a large potential of yet “undiscovered” use of natural resources. There are numerous examples for traditional knowledge of plant use as a starting point for the development of products used in modern societies, such as drugs, industrial resources or cosmetic products [6]. A large amount of yet undiscovered resources is to be expected in global plant diversity [7]. However, due to changes in human population structure and the decreasing interest of younger generations in traditional lifestyle, a considerable amount of the knowledge on traditional plant use is in danger of being lost [3, 4]. This effect is even increased by the influence of climate change and land use change leading to an increasing habitat loss for many used plant species. A clear, comprehensive scientific documentation of traditional plant use is thus an indispensable tool to preserve this valuable knowledge and the basis for a further sustainable use of biodiversity. Especially, an understanding of plant use in a larger spatial and plant-systematic context might help to focus future research effort and improve conservation strategies.

### Traditional medicine

The WHO estimates that up to 80% of the world’s population rely on traditional medicine (TM) for health care [8]. In many ethnic groups the use of plants and plant products in traditional medicine is one of the most important applications of plants [9]. Guinko [10] estimated that 90% of the population of BFA relied entirely on traditional remedies for health care. While these numbers seem to have decreased in the last 30 years, there is no doubt, that traditional medicine remains an important element in the Burkinabé society and a major source of medication for large parts of the population [2, 4, 11]. The application of plants as remedies is deeply anchored in the social structure of the communities in the country. A better knowledge of the plant use in TM and the validation of pharmacological effect using modern scientific approaches can thus benefit a large amount of people.

The link between plant use in TM and actual pharmacological activity has been subject of controversy. The use of a plant species in TM might be related to the presence of physiologically active phytochemical compounds, but might also be rather culturally motivated [12, 13]. However, it has been found that plants with long, effective use in traditional medicine are likely to have a pharmaceutical effect [6, 12, 14]. Indeed, numerous studies have given examples for the pharmacological activity of traditionally used plants [5, 8, 15]. A large number of drugs have their direct origin in phyto-pharmacological substances (e.g. Taxol, Aspirin, Artemisinin) and even synthetically developed drugs have been rediscovered naturally occurring in plants used in TM [16]. One indicator of pharmaceutical activity is the use of a species in different cultures or by different healers [12, 17]. Hence, analyses of plant use across multiple ethnical groups are a promising approach to identify plants containing pharmacologically active substances. This approach might be enhanced by linking data on plant use with systematic information on plant relationships. Phylogenetically closely related species are more likely to contain similar phytochemical compounds, and therefore a clustered use of species of one plant family in TM, or the application of closely related species as remedy against specific health disorders might be evidence for the presence of physiologically active phytochemicals [17, 18]. In short, large-scale analyses, integrating different ethnic groups and taking the phylogenetic relationship of plants into account are a powerful tool to identify promising species for drug screening [17, 19].

### Plant use and conservation

Burkina Faso is located in a region especially susceptible to climate change [20] and is likely to face severe environmental and socio-economic changes in the 21^st^ century. Expected population growth together with the influence of climate change on flora and vegetation creates a challenging situation for environmental conservation [21, 22]. The combination of environmental change and increasing exploitation pressure is especially critical for the conservation of useful plants [23, 24]. Detailed knowledge of use patterns, actual usefulness and especially pharmacological effectiveness are the base for effective conservation [25]. Furthermore, the presence of useful plants can be an important argument to local communities for conservation areas [26]. The inclusion of local communities into the conservation efforts has been shown to be crucial for sustainable conservation (e.g. [23]).

In the last 20 years there has been an intensification of ethnobotanical research in Burkina Faso [2–5, 7, 8, 11, 23–51]. However, a quantitative, national-scale analysis of plant use in the country was missing until now. We use a currently published plant checklist [52] and the underlying database together with data from multiple ethnobotanical studies of the region to present an overview of plant use in BFA with a focus on TM. Understanding the national patterns of plant use in BFA is highly relevant, as a detailed knowledge of traditional plant use can a) help to set conservation priorities by identifying species that are prone to overexploitation and b) help to communicate conservation effort to local communities by including species of high usefulness in conservation planning. Furthermore the results presented here on plant use in TM might help to focus research on pharmacological activity of plant derived remedies and thus benefit local communities and possible pharmacological screenings. Due to the relative homogeneity of flora and vegetation throughout dry West Africa, the results presented here for BFA might be considered representative for the much larger region of the West African savanna biome.

## Methods

Our analyses included all plant species known from BFA (including introduced species) [52]. The plant use information was based on 47 different references published between 1971 and 2014 [2–5, 7, 8, 10, 23, 24, 26, 27, 30, 31, 33–63]. These sources included ethnobotanical studies from Burkina Faso as well as information from floras of Burkina Faso and neighbouring countries. We included data from neighbouring countries, as the different ethnic groups of the Burkinabé population are also present in neighbouring countries, and the plant use is expected to be relatively homogenous within these groups. See Additional file 1 for a detailed information on the source material. A literature database compiled by the authors was completed with a literature search in the databases of PubMed and Web of Science using combinations of the keywords “Burkina Faso” and “plant use”, “useful plant”, “medicinal plant”, “ethnobotany”, “traditional medicine”, “medicinal plant”, “traditional plant use”, “ethnobotanique”, “plante utile”, “utilisation plante”, “plante médicinale” respectively. From the result we included studies that were based on ethnobotanical interviews in Burkina Faso and that reported unambiguous scientific plant names as base for our analyses (but see Additional file 2 for a list with vernacular names for the most common species). We explicitly excluded studies that were solely concerned with pharmacological screenings or agricultural practices as well as articles dealing only with one single species. The latter was done to avoid overweighting and refers to only a few economically important species that are well covered with the dataset. The African plant database [64] was used as reference for scientific plant names, and synonyms were included under their accepted name. We used keywords to categorize the detailed information from literature into ten plant use categories: construction, cultivation, firewood, fodder (animal nutrition), traditional medicine (TM), human nutrition, ornament, religion and art, tools and craft, veterinary. The categories were chosen to reflect the most common uses and are orientated on the level 1 and 2 states of the Economic Botany Data Collection Standard [65]. See Additional file 3 for a classification of each species to the 10 use categories.

To further investigate the use of plants in TM we classified the detailed medicinal information from the references into 22 health disorder categories. We followed the Economic Botany Data Collection Standard [65] for the classification. The classification-scheme was slightly modified to meet the local characteristics. Three categories were added: Child specific (i.e. all medication directed specifically to children or growth disorders), internal organs (including liver, spleen and kidney disorders) and oral/teeth (oral hygiene, oral and tooth disorders). Disorders related to the circulatory system and blood were combined to one category. We classified the plants using over 500 keywords and a subsequent visual check of each species description.

We used the number of references citing the use of a species and the number of use categories (see above) per species to calculate the relative importance index and to rank species according to their usefulness. The RI was calculated following [28]:


With: RFC = relative frequency of citation (Frequency of citation/Number of References), RNU = Relative number of use-categories (Number of uses/Maximum number of uses of a species).

## Results

**Figure 1:**
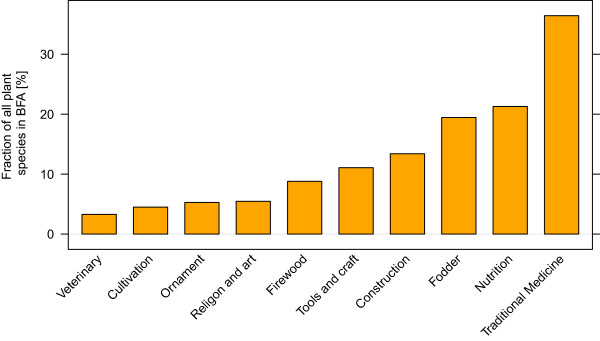
**The importance of different plant use categories in traditional plant use in Burkina Faso.** The bars represent the percentage of species of the total known flora (2067 species) used in ten different categories. The most species are used for traditional medicine, human nutrition and animal fodder.

Out of the 2067 known plant species of Burkina Faso 1033 (50%) had a traditional use recorded. Figure 1 shows the use of plant species in 10 different use categories. Most species were used for traditional medicine (36% of all species) followed by human nutrition (21%) and animal fodder (19%).

**Figure 2:**
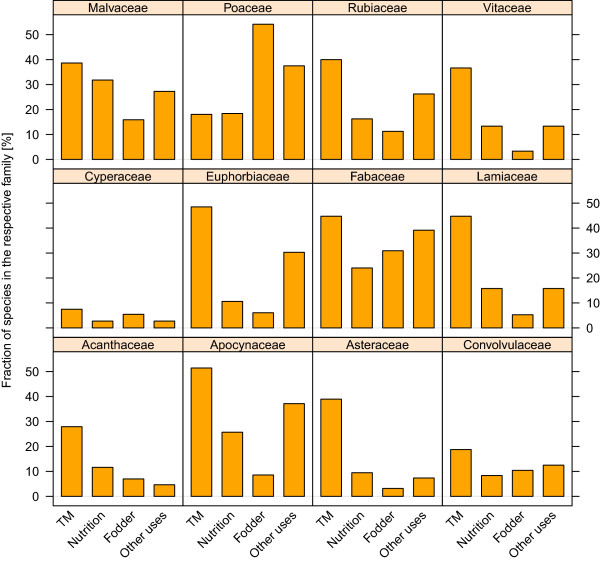
**The use spectrum of the twelve most species-rich plant families in Burkina Faso.** The bars represent the percentage of species in the respective family used in four different use categories. The three most important use categories (traditional medicine, human nutrition and animal fodder) as well as other uses are shown. Other uses include the use for construction, tools and crafts, firewood, ornament, veterinary as well as religion and art. The use patterns differ considerably. Large proportions of the Apocynaceae, Euphorbiaceae and Lamiaceae are used for medicine. Cyperaceae and Convulvulaceae are generally scarcely used.

The purpose of traditional use was highly related to plant family. Fabaceae, Poaceae and Malvaceae were the plant families with the most species relevant for traditional plant use. The twelve most species rich families in BFA differed with regard to the amount of species used and the purpose of use (Figure 2). While most of the families were employed in multiple categories, some families were only used for specific purposes. The two most species-rich families in the country, Fabaceae and Poaceae, were of special importance for human nutrition and animal fodder. Together they comprised 29% of all species used for human nutrition and approximately 62% of all plant species known to be used as fodder. The Fabaceae were also of special importance in TM, comprising 18% of all plants used in traditional medicine. Intriguingly, species of some families were rarely used in any way. Especially Cyperaceae and Convolvulaceae included only a low number of useful species (Figure 2).

Table 1 shows the 20 “top used” plant species in the country according to the relative importance index. *Khaya senegalensis, Adansonia digitata* and *Diospyros mespiliformis* were the top ranked species. The list includes five Fabaceae, two Malvaceae and two Combretaceae species. All species listed in Table 1 are woody plants. See Additional file 4 for a usefulness evaluation of every species with at least one known use in the country.

**Table 1:** **The 20 top useful plant species in Burkina Faso based on the relative importance index**

Accepted name	Family	Number of uses	Frequency of citation	Relative frequency of citation	Relative importance index
*Khaya senegalensis* (Desr.) A.Juss.	Meliaceae	9	24	0.5	0.98
*Adansonia digitata* L.	Malvaceae	8	25	0.52	0.944
*Diospyros mespiliformis* Hochst. ex A.DC.	Ebenaceae	8	25	0.52	0.944
*Vitellaria paradoxa* C.F.Gaertn.	Sapotaceae	8	25	0.52	0.944
*Balanites aegyptiaca* (L.) Delile	Zygophyllaceae	8	24	0.5	0.924
*Tamarindus indica* L.	Fabaceae	8	24	0.5	0.924
*Parkia biglobosa* (Jacq.) R.Br. ex G.Don	Fabaceae	8	22	0.46	0.884
*Mitragyna inermis* (Willd.) Kuntze	Rubiaceae	8	21	0.44	0.864
*Annona senegalensis* Pers.	Annonaceae	8	20	0.42	0.844
*Sclerocarya birrea* (A.Rich.) Hochst.	Anacardiaceae	8	20	0.42	0.844
*Pterocarpus erinaceus* Poir.	Fabaceae	8	19	0.4	0.824
*Anogeissus leiocarpa* (DC.) Guill. & Perr.	Combretaceae	7	21	0.44	0.809
*Guiera senegalensis* J.F.Gmel.	Combretaceae	7	21	0.44	0.809
*Lannea microcarpa* Engl. & K.Krause	Anacardiaceae	7	21	0.44	0.809
*Piliostigma reticulatum* (DC.) Hochst.	Fabaceae	8	18	0.38	0.804
*Detarium microcarpum* Guill. & Perr.	Fabaceae	7	20	0.42	0.789
*Combretum glutinosum* Perr. ex DC.	Combretaceae	8	17	0.35	0.784
*Ficus sycomorus* L.	Moraceae	7	19	0.4	0.769
*Sterculia setigera* Delile	Malvaceae	7	19	0.4	0.769
*Ximenia americana* L.	Ximeniaceae	7	19	0.4	0.769

Number of uses = Number of different uses of the species (from a total of 10 categories; see Figure 1); frequency of citation = number of references naming a use of this species. Relative importance index: calculation modified after [28] as described in the methods section.

### Traditional medicine

**Figure 3:**
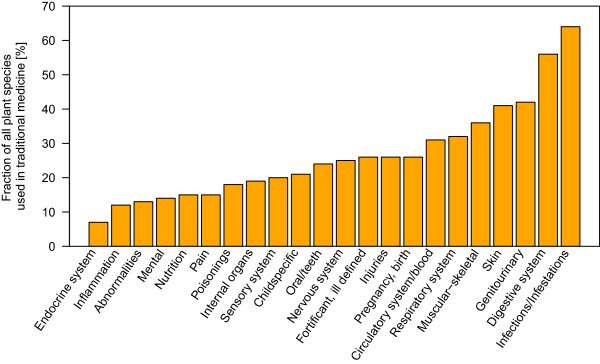
**Twenty-two different health disorders addressed with medicinal plants.** The bars represent the number of species applied as remedy for the respective disorder as percentage of all species used in traditional medicine (753 species). Often plants are used in multiple categories. “Infections/Infestations”, digestive system disorders and genitourinary disorders are the most commonly addressed health disorders. The categories are modified after [65].

**Figure 4:**
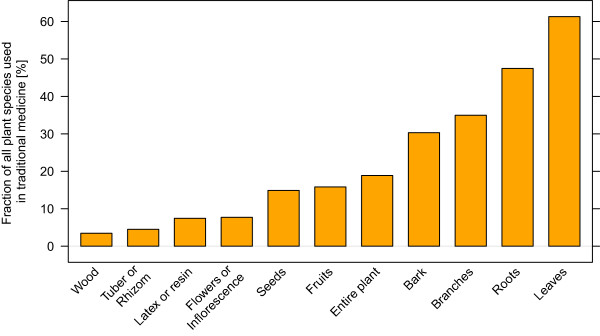
**The importance of different plant organs in traditional medicine.** The bars represent the number of species where the respective organ is used in TM as percentage of all species with a known use in TM (753 species). Often multiple plant parts are used per species. Leaves, roots and branches are the plant organs most commonly used in TM.

More than one third of the 2067 species known from BFA had a recorded use in TM (753 species). The biggest portion of the species was applied as remedy against disorders of the categories infections/infestations (64%), digestive system disorders (56%) and genitourinary disorders (42%). In the case of infections/infestations, malaria, icterus, worm parasites or sexual transmitted diseases were the most commonly targeted disorders. Figure 3 shows the number of plant species applied as remedies in 22 disorder categories. Over all, leaves and roots were the most commonly used plant parts (Figure 4).

**Figure 5:**
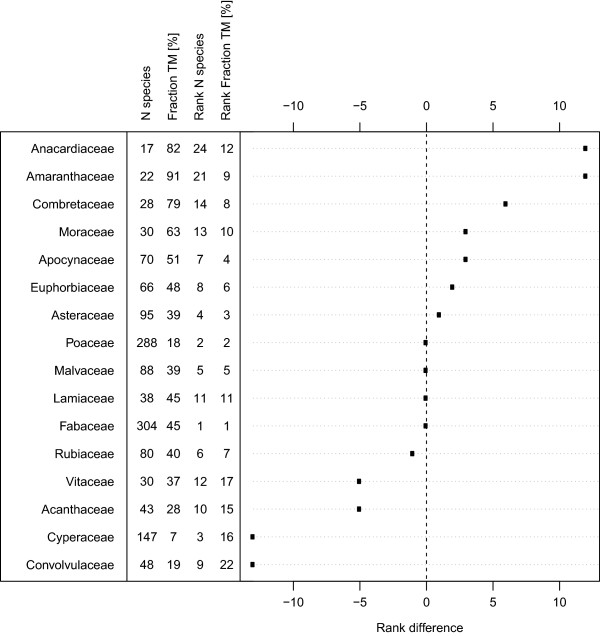
**The relative importance of plant families in traditional medicine in Burkina Faso.** The figure shows the difference between a family’s rank regarding total species number and its rank regarding number of species used in traditional medicine. N = total number of species, Fraction TM [%] = percentage of these species used in Traditional medicine, Rank N species = Rank of the family regarding total species number in the country, Rank Fraction TM = rank of the family regarding species used in traditional medicine. The listed families comprise the 12 most species rich families in the country and the 12 plant families most commonly used in TM. Anacardiaceae, Amaranthaceae and Combretaceae are relatively over-used, Convolvulaceae, Cyperaceae, Acanthaceae and Vitaceae are relatively under-used.

On a broader systematic scale, species of Anacardiaceae, Amaranthaceae, Combretaceae and Moraceae were over-proportionally used in traditional medicine compared to the families’ species richness in BFA (Figure 5). In contrast, species of Convolvulaceae, Cyperaceae, Acanthaceae and Vitaceae were under-proportionally used. Corresponding to the list of the twenty top useful plants (Table 1), we calculated the RI including only medicinal use to rank all plant species in BFA according to their importance in TM (Table 2). *Tamarindus indica, Vitellaria paradoxa* and *Adansonia digitata* were the top used species (see Additional file 5 for a ranking including all species with at least one known use). All species in Table 2 are woody plants.

**Table 2:** **The 20 top useful medicinal plants in Burkina Faso based on the relative importance index**

Accepted name	Family	Number medicinal of uses	Frequency of citation	Relative frequency of citation	Relative importance index
*Tamarindus indica* L.	Fabaceae	18	21	0.53	0.974
*Vitellaria paradoxa* C.F.Gaertn.	Sapotaceae	17	19	0.48	0.9
*Adansonia digitata* L.	Malvaceae	19	16	0.4	0.881
*Ximenia americana* L.	Ximeniaceae	18	17	0.43	0.878
*Khaya senegalensis* (Desr.) A.Juss.	Meliaceae	16	19	0.48	0.873
*Diospyros mespiliformis* Hochst. ex A.DC.	Ebenaceae	16	18	0.45	0.85
*Lannea microcarpa* Engl. & K.Krause	Anacardiaceae	16	18	0.45	0.85
*Annona senegalensis* Pers.	Annonaceae	18	15	0.38	0.831
*Ficus sycomorus* L.	Moraceae	16	17	0.43	0.826
*Combretum micranthum* G.Don	Combretaceae	18	14	0.35	0.807
*Sterculia setigera* Delile	Malvaceae	17	15	0.38	0.805
*Balanites aegyptiaca* (L.) Delile	Zygophyllaceae	16	16	0.4	0.802
*Lannea acida* A.Rich.	Anacardiaceae	17	14	0.35	0.781
*Guiera senegalensis* J.F.Gmel.	Combretaceae	16	15	0.38	0.778
*Pterocarpus erinaceus* Poir.	Fabaceae	16	15	0.38	0.778
*Parkia biglobosa* (Jacq.) R.Br. ex G.Don	Fabaceae	15	16	0.4	0.776
*Sarcocephalus latifolius* (Sm.) E.A.Bruce	Rubiaceae	15	16	0.4	0.776
*Cassia sieberiana* DC.	Fabaceae	14	17	0.43	0.773
*Entada africana* Guill. & Perr.	Fabaceae	18	12	0.3	0.759
*Sclerocarya birrea* (A.Rich.) Hochst.	Anacardiaceae	16	13	0.33	0.731

Number of medicinal uses = Number of different health disorders addressed (from a total of 22 categories, see Figure 3); frequency of citation = number of references naming a use of this species.

## Discussion

We found a clear phylogenetic clustering of plant use. The fact that species of some families are preferentially used for specific purposes is an often recorded fact and has been attributed to specific traits more common in these families [3, 17]. For example the preferential use of Poaceae and Fabaceae species for human and animal nutrition can be related to the often nutritious fruits or seeds in these families.

In the case of TM the phylogenetic clustering might well be related to the presence of phytochemical compounds [5, 8, 15]. Inversely, the relative sparse use of Cyperaceae species has been related to the relative low content in phytochemicals in this family [3]. Interestingly, many plant species are applied as remedy only in few health disorder categories (Additional file 6). While the general link between plant use and pharmacological activity is debated controversially, the clear phylogenetic pattern and the specific use of most species shown here might help to guide drug screenings.

The fact that more than one third of all plant species of BFA have a known medicinal use stresses the importance of TM for the population, especially in the rural communities, of the country. The high number of medicinal plants used to address infections/infestations, digestive system disorders and genitourinary disorders is a clear indication of the importance of these disorders in the country. Especially digestive system disorders are documented to be specifically common in West Africa [12]. The identification of malaria, icterus and gastro-intestinal disorders as main targets for traditional medicine is consistent with other studies and is most likely related to the high number of infections and the importance of these diseases in the people’s lifes [6, 12, 26]. Malaria and malaria related symptoms were by far the most targeted diseases in this study, which accounts for the large number of malaria cases in BFA. Malaria is a major threat to the people in the country, with 3.5 million recorded cases in 2008 (thereof 50% among children under 5 years [66]) and has been reported as a main target for traditional medicine in BFA [11]. At the same time malaria is an example for the successful use of natural products and traditional medicine to guide drug screening and development [5, 6, 8, 67]. This is of special importance, as resistance against commonly used drugs is becoming a severe challenge for malaria treatment in the region [68].

The “top usefulness” rankings of plant species (Table 1 and Table 2) are the first comprehensive assessment of this type on a national scale. Generally our rankings were successful in identifying plants of known high importance, and agree well with local scale assessments. Ten of the species shown in Table 1 (*Adansonia digitata*, *Diospyros mespiliformis*, *Vitellaria paradoxa*, *Balanites aegyptiaca*, *Tamarindus indica*, *Parkia biglobosa*, *Annona senegalensis*, *Sclerocarya birrea*, *Detarium microcarpum* and *Ximenia americana*) were identified as important plants in the traditional agroforestry systems of the Sudanian zone in Benin [69]. In another study nine species from Table 1 (*Khaya senegalensis*, *A. digitata*, *D. mespiliformis*, *V. paradoxa*, *T. indica*, *P. biglobosa*, *Pterocarpus erinaceus*, *Anogeissus leiocarpa* and *D. microcarpum*) were ranked within the thirty most important woody plant species across multiple ethnic groups and multiple use categories in Northern Benin [70]. The same study includes seven of our twenty top useful medicinal plants (Table 2) in a list of the most important medicinal plants in this area (*T. indica, V. paradoxa, A. digitata*, *K. senegalensis, P. erinaceus, Sarcocephalus latifolius* and *Entada africana).* A third study identified *A. digitata, V. paradoxa, T. indica, D. microcarpum* and *P. biglobosa* as key species for plant use of the Gourounsi people in central BFA [36]*.* A study in the Pendjari Biosphere Reserve in Benin evaluating non-timber forest products agreed in ranking eight of the top 20 species presented here in a list of the 15 most important used species (*K. senegalensis, A. digitata, D. mespiliformis, V. paradoxa, T. indica, P. biglobosa, L. microcarpa* and *Ficus sycomorus)*[71]. Of course, these results must be interpreted carefully. Some species identified as commercially important in other studies were not ranked as top use species in our list (especially *Vitex doniana)*. This might be explained by a rather focused use (and thus a lower relative importance index). See Additional file 4 for a usefulness ranking list including more species. Additionally, the ranking is depending on the reference studies used to build the database and the characteristics of the relative importance index*.* While a high number of studies mentioning use of a species and a large number of different use categories can be interpreted as indicator of species importance, a low number of uses or references does not necessarily mean that a species is not of high value for specific purposes or on a local scale [3]. Generally, the ranking should be understood as a tool to identify a set of key species with a relative high use value across the country. Identifying such species is an important prerequisite for conservation planning [36].

Plant use highly depends on social factors and differs considerably between different ethnic groups and locations. Interview-based studies are a key to understanding and preserving traditional ethnobotanical knowledge. However, in times of climate change, when large scale conservation strategies are urgently needed, large-scale analyses of plant use are equally necessary. Including key economic species for local communities into conservation planning can highly increase the success of these efforts and make sure that they benefit as many people as possible.

## Conclusions

We revealed a clear systematic pattern of traditional plant use throughout BFA, and identified the importance of specific plant families for specific uses. This systematic pattern is especially interesting in the context of plant use in traditional medicine, as it might correlate with pharmacological activity. The evaluation of usefulness of each plant species using the relative importance index has provided a robust hit list of the “top useful” species in the country and will be an important tool in focussing future conservation effort and possibly pharmacological screening. Our results are of interest for applied research, as a detailed knowledge of traditional plant use can a) help to communicate conservation needs and b) facilitate future research on drug screening.

## Electronic supplementary material

Additional file 1: **Detailed references of plant use information.** (XLSX 19 KB)

Additional file 2: **List of vernacular names in Mòoré and scientific names of the most common plants.** (XLSX 23 KB)

Additional file 3: **Detailed use information on each species.** (XLSX 136 KB)

Additional file 4: **Usefulness ranking of all species with at least one documented use.** (XLSX 67 KB)

Additional file 5: **Usefulness ranking in traditional medicine of all species with at least one documented use.** (XLSX 56 KB)

Additional file 6: **Histogram of the number of applications in traditional medicine per species.** (PDF 2 KB)
