# Supplementary figures and images for: Traditional plant use in Burkina Faso (West Africa): a national-scale analysis with focus on traditional medicine
Source: J Ethnobiol Ethnomed. 2015 Feb 19;11:9. doi: 10.1186/1746-4269-11-9 (PMC4429461; doi:10.1186/1746-4269-11-9)

**Histogram of the number of medicinal applications per species**

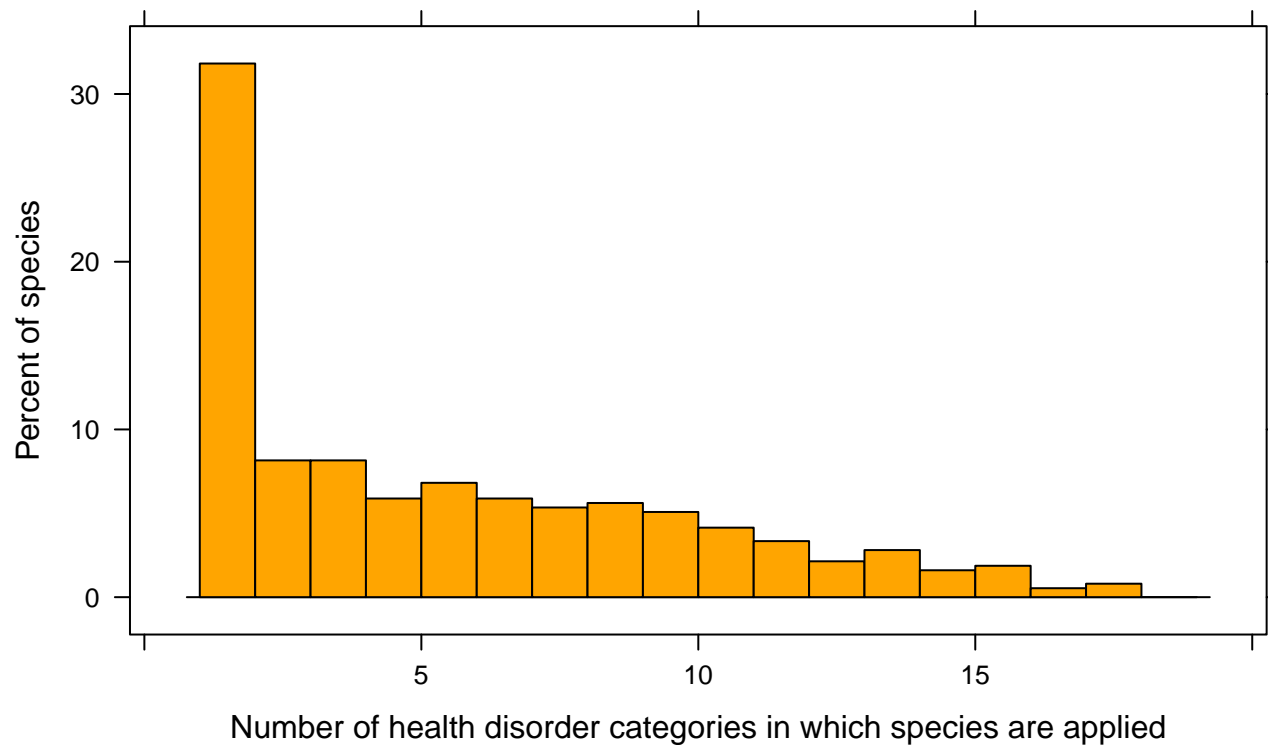

Supplement: Supplementary file 6 — Additional file 6: Histogram of the number of applications in traditional medicine per species. (PDF 2 KB) [file 13002_2014_473_MOESM6_ESM.pdf]
